# Pre-Microporation Improves Outcome of Pancreatic Islet Labelling for Optical and ^19^F MR Imaging

**DOI:** 10.1186/s12575-017-0055-4

**Published:** 2017-06-28

**Authors:** Vít Herynek, Andrea Gálisová, Mangala Srinivas, Eric A. W. van Dinther, Lucie Kosinová, Jiri Ruzicka, Markéta Jirátová, Jan Kriz, Daniel Jirák

**Affiliations:** 1 MR Unit, Radiodiagnostic and Interventional Radiology Department, Institute for Clinical and Experimental Medicine, Vídeňská 1958/9, Prague, Czech Republic; 2 Department of Tumor Immunology, Radboud University Medical Centre, Route 278, Geert Grooteplein 28, Nijmegen, Netherlands; 3 Centre of Experimental Medicine, Institute for Clinical and Experimental Medicine, Vídeňská 1958/9, Prague, Czech Republic; 4 Department of Tissue Culture and Stem Cells, Institute of Experimental Medicine AS CR, Vídeňská 1083, 142 20, Prague, Czech Republic; 5 Diabetes Centre, Institute for Clinical and Experimental Medicine, Vídeňská 1958/9, Prague, Czech Republic

**Keywords:** Pancreatic islets, Cell labelling, Microporation, Endocytosis, Bimodal nanoparticles, ^19^F magnetic resonance imaging, Fluorescence imaging, Confocal microscopy

## Abstract

**Background:**

In vitro labelling of cells and small cell structures is a necessary step before in vivo monitoring of grafts. We modified and optimised a procedure for pancreatic islet labelling using bimodal positively charged poly(lactic-co-glycolic acid) nanoparticles with encapsulated perfluoro crown ethers and indocyanine green dye via microporation and compared the method with passive endocytosis.

**Results:**

Pancreatic islets were microporated using two pulses at various voltages. We tested a standard procedure (poration in the presence of nanoparticles) and a modified protocol (pre-microporation in a buffer only, and subsequent islet incubation with nanoparticles on ice for 10 min).

We compared islet labelling by microporation with labelling by endocytosis, i.e. pancreatic islets were incubated for 24 h in a medium with suspended nanoparticles.

In order to verify the efficiency of the labelling procedures, we used ^19^F magnetic resonance imaging, optical fluorescence imaging and confocal microscopy.

The experiment confirmed that microporation, albeit fast and effective, is invasive and may cause substantial harm to islets. To achieve sufficient poration and to minimise the reduction of viability, the electric field should be set at 20 kV/m (two pulses, 20 ms each).

Poration in the presence of nanoparticles was found to be unsuitable for the nanoparticles used. The water suspension of nanoparticles (which served as a surfactant) was slightly foamy and microbubbles in the suspension were responsible for sparks causing the destruction of islets during poration. However, pre-microporation (poration of islets in a buffer only) followed by 10-min incubation with nanoparticles was safer.

**Conclusions:**

For labelling of pancreatic islets using poly(lactic-co-glycolic acid) nanoparticles, the modified microporation procedure with low voltage was found to be safer than the standard microporation procedure. The modified procedure was fast, however, efficiency was lower compared to endocytosis.

## Background

Organ transplantations are widely used as therapeutic procedures for various diseases. However, due to limited organ availability, immunological problems and substantial organ impairment during surgery or cold ischemia, cell transplantations have now become a focus of biomedical research [1]. In several areas, such as the transplantation of haematopoietic stem cells [2] and pancreatic islets [3, 4], procedures have been established in clinical practice to treat both leukaemia and Type 1 diabetes mellitus, respectively. Nevertheless, cell (or small tissue structure) transplantations are, even after 20 years of research, still at a developmental stage and further research tools are required in order to monitor transplantation outcomes. Of the various in vivo imaging methods for imaging transplanted cells, most require cells and cell structures to be suitably labelled [5].

Labels may bear the following: a radionuclide for positron emission tomography (PET) and single-photon emission computed tomography (SPECT); a fluorescent dye for optical imaging; a paramagnetic or superparamagnetic core for ^1^H magnetic resonance imaging (MRI); a fluorine compound for ^19^F MRI. Labels and detection methods differ with regard to the sensitivity, specificity and accuracy of spatial localisation. Sensitive PET and SPECT lack sufficient spatial resolution [6], ^1^H MRI at high spatial resolution is not specific [7], specific ^19^F MRI lacks sensitivity [8] and the sensitivity of optical imaging substantially decreases with distance from the surface [9]. Therefore, in order to take advantage of several imaging methods, bi- or multi-modal labels are often used [5].

Cell labelling using ^19^F tracers was reported for the first time by Ahrens et al. [10]. Various labels containing fluorinated compounds have been successfully used for in vitro labelling and in vivo tracking of stem cells [11, 12] or immune cells [13, 14], and were even used in vivo for human mononuclear cells [15] in an animal model. Moreover, ^19^F tracers can be used for quantification of cells in vivo [13, 16]. In addition, combination of two types of labels may be advantageous for monitoring of two distinct cell populations or their interaction [17].

Various labelling procedures are used for different imaging modalities [18]: simple endocytosis [7], enhanced endocytosis using a suitable transfection agent [19], labelling using a specific antigen [20] and electro- or micro-poration [21]. Similar procedures used for cell labelling can be adapted for labelling pancreatic islets (PIs) [22] and imaging strategies can be combined [23]. In addition to the most common labelling procedure which uses endocytosed iron oxide nanoparticles, particles can be bound to islet surfaces [24] or gadolinium-based paramagnetic labels can be used [25]. The influence of positively charged nanoparticles upon uptake has also been reported [26].

Microporation (electroporation in a capilary using a higher voltage) was introduced by Kim et al. [27] and Lim et al. [28] although they used the method for gene delivery into the cells. Electroporation for labelling of PIs was used for the first time by Foster et al. [29].

Labelling pancreatic islets is particularly difficult due to their structure [30, 31]. Highly vascularized organs consist of endocrine cells secreteing insuline (β-cells), glucagon (α-cells), somatostatin (δ-cells), pancreatic-polypeptide (PP-cells), and ghrelin (ε-cells). The structure differs among species; for example, rodent islets broadly used in experiments have defined β-cell core surrounded by α-cells and other endocrine cells in the periphery. Human islets have endocrine cells more scattered (with higher number of α-cells). Different architecture influences islet function and its sensitivity to low glucose concentrations. Labelling procedures therefore may affect different cells in the case of animal and human islets, which should be kept in mind when translating the experiments to clinical practice. To avoid usage of transfection agents and to keep the labelling system simple, a positive charge added to the label surface may increase cell labelling efficiency [26].

Although electroporation was used for pancreatic islet labelling by nanoparticles [32], microporation described by Kim et al. [27] is used mostly for gene delivery into stem cells [28] or into pancreatic islets [33], or for labelling of islets by small molecules [34].

In this study, we optimised the parameters of a microporation procedure for labelling rat PIs using positive-charged bimodal nanoparticles for ^19^F MRI and optical imaging. The procedure was modified to improve labelling outcomes in comparison with endocytic labelling.

## Methods

### Nanoparticles

Poly(lactic-co-glycolic acid) (PLGA) nanoparticles with entrapped perfluoro-15-crown-5-ether (PFCE; for ^19^F MRI) and indocyanine green (ICG; for fluorescence optical imaging) were prepared using an o/w emulsion and solvent evaporation-extraction method as described previously [35]. Briefly, 200 mg of PLGA (Resomer RG 502 H, lactide/glycolide molar ratio 48:52 to 52:48; Boehringer Ingelheim, Germany) in 6 mL of dichloromethane, containing 1800 μL PFCE (Exfluor Inc., Round Rock, TX, USA) and 100 μL ICG-PULSION solution (10 mg/mL) (PULSION Medical Inc., Feldkirchen, Germany) was added dropwise to 50 ml of aqueous 0.4% polyvinyl alcohol and 1.6% diethylaminoethyl-dextran and emulsified for 120 s using a digital sonicator (Branson Ultrasonics, Danbury, CT, USA). The solvent was evaporated and nanoparticles were collected by centrifugation at 14.000 rpm for 20 min, washed six times with distilled water and lyophilised. Dynamic light scattering (DLS) was performed on a Malvern Zetasizer Nano (Malvern Instruments Ltd., Malvern, United Kingdom). Sizes varied in the range of 210–360 nm and zeta potentials were 6–27 mV. PFCE content was measured on a Bruker Avance III 400 MHz NMR (Bruker, Rheinstetten, Germany). Nanoparticles contained 2.7–6.0×10^18^ F atoms/mg dry weight.

### Rat PI Isolation

We used pancreatic islets from Brown-Norway and Lewis rats. Pancreatic islets were isolated according to a protocol described by Gotoh [36]. Briefly, collagenase (1 mg/ml; Sevapharma, Prague, Czech Republic) was injected intraductally, after which the distended pancreas was excised and gently shaken at 37 °C for 20 min. Islets were separated from exocrine tissue using centrifugation in a discontinuous Ficoll® gradient (Sigma-Aldrich, St. Louis, MO, USA). Isolated islets were cultured for 24 h (37 °C, 5% CO_2_ atmosphere) in a CMRL-1066 medium (PANBiotech GmbH, Aidenbach, Bavaria, Germany) supplemented with 10% foetal bovine serum (FBS), 5% HEPES and 1% penicillin/streptomycin/L-glutamine (all Sigma-Aldrich).

### PI Labelling

#### Endocytosis

Endocytosis is a form of active transport used by cells to internalise large polar molecules or solid particles, which cannot pass through the hydrophobic plasma membrane. In this energy-requiring process, used as a means of a feeding, the cell engulfs the particle by forming a membrane vesicle. First, plasma membrane creates a small invagination where the particle is captured. Then, the particle is fully surrounded forming a membrane vesicle carrying the captured substance inside. Because of the negative charge of the cell surface, positively charged nanoparticles increase efficiency of the transfection [37].

Two hundred and fifty isolated PIs were incubated at 37 °C in a medium (84% CMRL-1066 medium, 10% FBS, 5% HEPES, 0.5% penicillin/streptomycin, and 0.5% glutaMAX (ThermoFisher Scientific, Waltham, MA, USA)) with suspended nanoparticles. The concentration of the nanoparticles was 23 mg/mL medium. After 24-h incubation, pancreatic islets were collected, washed three times using phosphate buffer saline (PBS) and counted.

#### Microporation – Standard Procedure

Microporation – a modified electroporation method - is a microbiology technique in which an electrical field is applied to cells in order to temporarily increase the permeability of the cell membrane, allowing labels, drugs, nucleic acids to be introduced into the cells. Microporation uses a pipette tip as an electroporation space and a gold-coated electrode surface, therefore a uniform electric field is produced with minimal heat production, metal ion dissolution, or oxide formation, which may impair cells during electroporation [38]. The standard protocol was based on protocols provided by the manufacturer of the device (see further) and tests performed by Lefebvre et al. [33]; according to all published protocols, the electrical pulses are applied in the presence of the substance of interest in the transfection buffer (although number of pulses, their voltage and length may differ).

The electroporation device Neon Transfection System (ThermoFisher Scientific, Waltham, MA, USA) was used for the experiment. Buffers from the original Neon™ Transfection System 100 μL Kit (ThermoFisher Scientific, Waltham, MA, USA) were used. The Neon Tube was filled by 3 mL of electrolytic Buffer E2 and the tube was placed into the Neon Pipette Station. One hundred and ten microliters samples containing 250 pancreatic islets resuspended in the Buffer R with suspended nanoparticles (the final nanoparticle concentration was the same at 23 mg/mL medium) were prepared. The islets were aspired into a 100 μL pipette tip and placed in the Neon Tube with the Electrolytic Buffer E2 according to the manufacturer’s instructions. The islets were then microporated using one, two, or four 20 ms pulses. The pulse voltage varied in the range 600–1500 V (electric field 20–50 kV/m). After microporation, the pipette was immediately removed and the microporated islets were placed in wells and kept on ice for 10 min. The islets were then collected and placed in Petri dishes containing 3 mL of medium without antibiotics and incubated at 37 °C for 24 h. After incubation, pancreatic islets were collected, washed three times using PBS, hand-picked and counted under a microscope.

#### Microporation – Modified Protocol – “pre-Microporation”

Contrary to the standard protocol, the pancreatic islets were porated without the nanoparticles. After this pre-microporation, the islets were subsequently incubated with the nanoparticles. This represents a fully new approach in terms of transfection of pancreatic islets.

The same device and chemicals were used as for the standard procedure. The Neon Tube was filled by 3 mL of electrolytic Buffer E2 and placed into the Neon Pipette Station. Wells in a 96-well plate were filled by 110 μL of medium with suspended nanoparticles (concentration 46 mg/mL). One hundred and ten microliters samples containing 250 pancreatic islets in the pure Buffer R were prepared. The islets were aspired into a 100 μL pipette tip and placed in a tube with the Electrolytic Buffer E2 according to the manufacturer’s instructions. The islets were then microporated using two 20 ms pulses. The pulse voltage was 500–1000 V (electric field 16.5–33 kV/m). After microporation, the samples were immediately placed in wells containing 110 μL of the medium with suspended nanoparticles. Final nanoparticle concentration after addition of the buffer containing pre-microporated islets was 23 mg/mL. The islets in the medium with nanoparticles were kept on ice for 10 min. The islets were then collected and placed in Petri dishes containing 3 mL of medium without antibiotics and incubated at 37 °C for 24 h. After incubation, pancreatic islets were collected, washed three times using PBS, hand-picked and counted under a microscope.

**Fig. 1:**
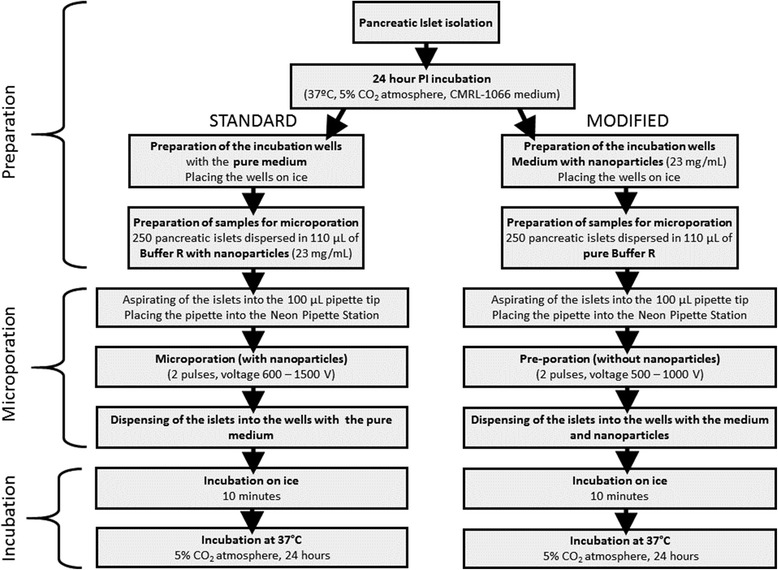
A flowchart describing the steps of the standard microporation (*left*) and modified pre-microporation (*right*) procedures

A chart in Fig. 1 shows the basic difference between the standard microporation procedure and the modified protocol utilising pre-microporation.

### Viability Test

After incubation, a viability test based on cell membrane integrity detection stained with acridine orange and propidium iodide [39] was performed. Acridine orange permeates both live and dead cells and stains all nucleated cells to generate a green fluorescence. Propidium iodide enters dead cells with poor membrane integrity and generates a red fluorescence. Cells stained with both dyes fluoresce red due to quenching, i.e. all live nucleated cells fluoresce green whereas all dead nucleated cells fluoresce red.

Ten islets in 20 μL of PBS were dropped into a 20 μL staining solution containing acridine orange (75 μM) and propidium iodide (9.4 μM) and then mixed. After 5 min of incubation, 250 μL of PBS was added and the islets were then inspected under a fluorescence microscope. Viability scores were determined according to the percentage of cells stained green (live) and red (dead) for each islet. The average percentage of viable cells was determined for each sample.

### Optical Fluorescence Imaging

Fluorescence images were acquired immediately after labelling on live islets in the medium placed in 0.5 mL test tubes using an IVIS Lumina XR imager (Perkin Elmer, Waltham, MA, USA) with the following parameters: exposure time 2 s, aperture four, excitation at 745 nm, emission filter at 810–875 nm. Regions of interest (ROI) of the same size were drawn over each sample. The optical signal from the ROI (average radiance) was expressed in arbritrary units normalized to the signal of islets labelled by endocytosis. Data were compared to the signal of PIs after endocytosis, which was performed with each microporation experiment (and was considered as a standard). Data could not be directly compared between different experiments due to possible variations in geometry setting. A standard photograph in the visible part of the light spectrum was acquired for co-registration of the optical signal.

### MR Imaging

After optical imaging, the pancreatic islets were fixed by formaldehyde (4%, Sigma-Aldrich) and placed in 0.5 mL test tubes prior to MR imaging. All MR measurements were performed using a 4.7 T Bruker BioSpin imager (Bruker, Rheinstetten, Germany) equipped with a homemade ^1^H/^19^F surface single-loop coil. The protocol consisted of an ^1^H MRI pilot scan and an ^1^H T_2_-weighted turbo-spin echo sequence (echo spacing TE = 12 ms, effective echo time TE_eff_ = 36 ms, repetition time TR = 3000 ms, turbofactor 8, number of acquisitions NA = 4, field of view FOV = 40 × 40 mm^2^, matrix 256 × 256). The coil was then tuned to ^19^F nuclei, and the frequency and transmitter setting was performed using a simple FID sequence. ^19^F MR images were acquired using a turbospin echo sequence (TE = 3.2 ms, TE_eff_ = 42.2 ms, TR = 1000 ms, turbo factor 32, NA = 4096, FOV = 40 × 40 mm^2^, matrix 32 × 32 interpolated to 256 × 256 to match the ^1^H images). Fluorine images were coloured red and superimposed over the ^1^H images in the gray scale by using ImageJ software [40].

### Confocal Microscopy

Pellets of the pancreatic islets were fixed using formaldehyde (4%, Sigma-Aldrich) overnight. The islets were then washed using PBS. Pellets were centrifuged (1 min at 1300 rpm), the supernatant was removed and agarose (2%, Sigma-Aldrich) was added. Pellets in the agarose were immediately centrifuged (1 min at 1800 rpm). After the agarose solidified, the pellets were transferred into sucrose (30%, Sigma-Aldrich) for overnight incubation at 4 °C. After incubation, the islets were transferred to Tissue-Tek (Sakura, Alphen aan den Rijn, Netherlands) and frozen in methylbutane (Sigma-Aldrich, St. Louis, MO, USA) cooled by liquid nitrogen. Frozen pellets were stored at −80 °C. Sections (20 μm) from the pancreatic islet pellets were cut using a cryomicrotome (Leica CM1950). The samples were stained with diamino-phenylindole (DAPI, Sigma-Aldrich, St. Louis, MO, USA) and mounted with a vectashield (Vector H-1000, Burlingame, CA, USA) on a glass slide. For confirmation of the nanoparticle signal and its location, the Olympus FV1200MPE (Olympus life Science, Tokyo, Japan) confocal microscope was used (green background - Argon laser λ = 488 nm, DAPI - EPI lamp λ = 405 nm, ICG - LD599 laser λ = 647 nm). The images were taken using 20× (air) and 60× (oil immersion) objectives under 200× or 600× magnification respectively.

### Statistical Analysis

Values in the graphs are presented as averages, error bars indicate standard deviations. Statistical tests were used for comparing the viabilities and gains of the differently treated samples containing pancreatic islets. As the data sets were small and did not have normal distribution, a nonparametric Mann-Whitney *U* test was employed. *P* < 0.05 was considered to indicate a statistically significant difference. Data from imaging methods were usually obtained from a limited number of samples (up to four), which did not allow meaningful usage of the statistical tests, therefore, average values with standard deviations only are provided. Further repetitive measurements were not performed due to ethical reasons; pancreatic islets cannot be reproduced in vitro like cell lines and each additional repetition would have required another group of 20 animals for PI isolation.

Number of pulses was optimised at one voltage value only, Mann-Whitney *U* test was used for comparison of both viability and imaging.

All animal experimental protocols were approved by the Ethics Committee of the Institute for Clinical and Experimental Medicine and the Ministry of Health of the Czech Republic in accordance with European Communities Council Directive 86/609/EEC.

## Results

### Microporation According to the Standard Procedure

At first, we optimised number of pulses used for microporation.

**Fig. 2:**
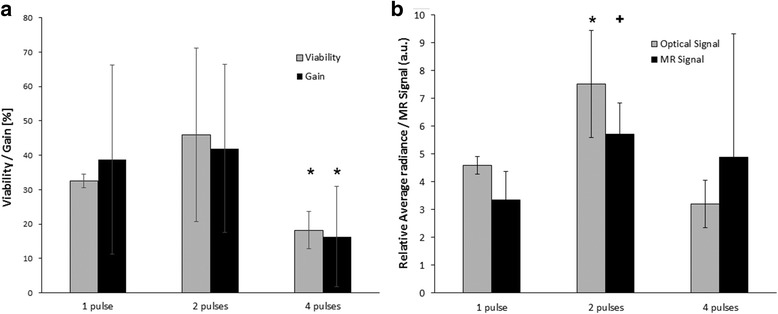
Comparison of viability, gain (**a**) and fluorescent and MRI signals (**b**) at various pulse numbers. Viability and gain were comparable at one and two pulses and significantly lower at four pulses (Mann-Whitney *U* test, *P* < 0.05, marked by “*”). Labelling efficiency was highest using two pulses; fluorescent signal was significantly higher than the signal of samples porated using one or four pulses (marked by “*”). MRI signal was significantly higher compared to the case of one pulse (marked by “+”) and comparable to the sample porated using four pulses

We compared viability, number of harvested islets (gain) and labelling efficiency after one, two and four pulses (Fig. 2). The experiment revealed similar viability and gain in the case of one and two pulses; both quantities significantly decreased when four pulses were used (Mann-Whitney *U* test, *P* < 0.05), see Fig. 2a. Labelling efficiency was highest in the case of two pulses (revealed by optical fluorescence imaging; MRI provided significantly lower signal at one pulse and similar signal at two and four pulses, see Fig. 2b). Therefore, for further experiments, we used two pulses only.

**Fig. 3:**
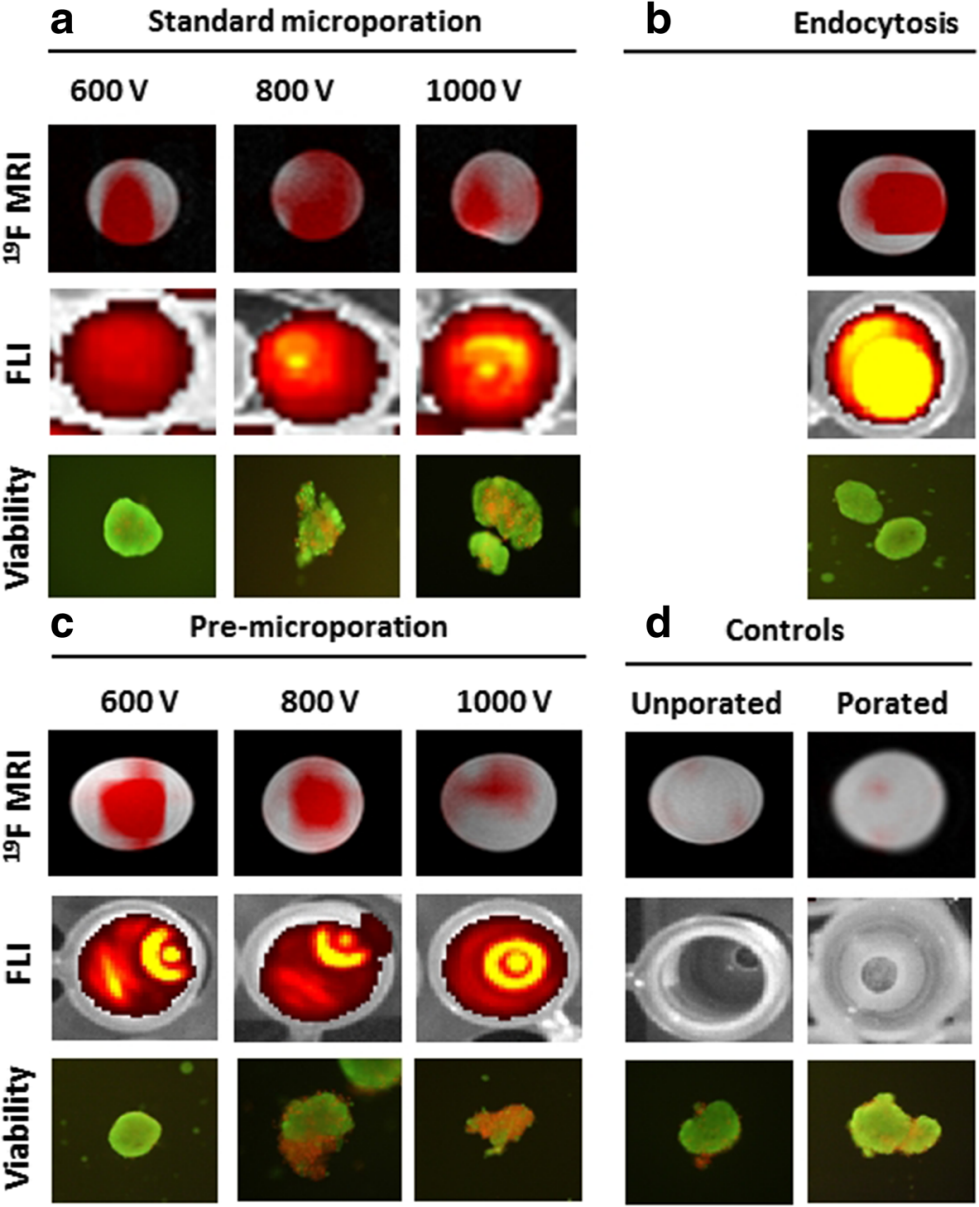
Images of pancreatic islets labelled with fluorine nanoparticles using microporation according to the standard protocol (**a**), endocytosis (**b**), pre-microporation (**c**), and unlabelled controls (**d**). ^19^F (*red*) MR images superimposed on ^1^H (*grey scale*) MR images (*upper row*), fluorescence images (*middle*) and selected microphotographs of islets stained for the viability test. Selected voltages (600, 800, 1000 V) used for microporation or pre-poration are shown. Unporated control represents unlabelled islets incubated without any intervention in the pure medium. Porated control contains islets porated in the buffer only (600 V) and incubated without nanoparticles

**Fig. 4:**
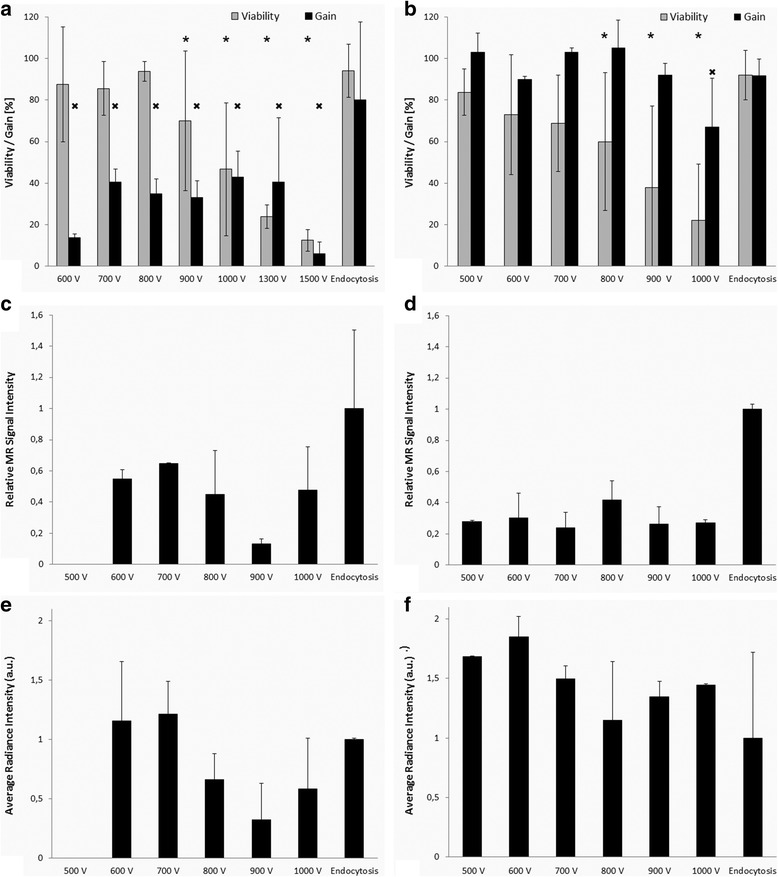
Viability and gain of pancreatic islets (**a**, **b**), MR signal (**c**, **d**) and fluorescent signal (**e**, **f**) from optical fluorescence imaging of phantoms containing PIs labelled by microporation using the standard procedure (*left*: **a**, **c**, **e**) and modified protocol (*right*: **b**, **d**, **f**). MR signal (**c**, **d**) was expressed as a contrast-to-noise ratio, fluorescent signal intensity is in arbitrary units proportional to average radiance. Significant decrease of viability (*P* < 0.05, marked by “*”) compared to endocytosis was observed at voltage 900 V and higher in the case of the standard protocol (**a**) and 800 V and higher in the case of modified protocol (**b**). Gain was substantially lower (*P* < 0.05, marked by “×”) in the case of the standard protocol at any voltage setting (**a**); whereas in the case of the modified protocol (**b**), significant gain decrease was observed at 1000 V only (*P* < 0.05, marked by “×”)

The representative ^19^F MRI, optical and viability images of PIs labelled using a standard microporation procedure, modified one (only selected voltages are presented), and endocytosis are shown in Fig. 3 including two control samples (unlabelled and unporated islets, and unlabelled islets after poration without nanoparticles). Figure 3 confirmed efficient labelling using the three labelling procedures, however, for proper comparison, MRI and optical signals, and viability needed to be properly quantified. Figure 4 summarised comparison of the standard protocol (Fig. 4a, c, e) and the modified one (pre-microporation, Fig. 3b, d,) by means of viability and gain of harvested islets (Fig. 4a, b), ^19^F MR signal (Fig. 4c, d), and fluorescent signal (Fig. 4e, f).

The percentage of harvested pancreatic islets 24 h after microporation according to the standard protocol and viability of harvested islets are shown in Fig. 4a, same data for modified protocol (pre-microporation) in Fig. 4b. Viability of the harvested islets was comparable for both methods. Viability of pancreatic islets microporated or pre-microporated at a lower voltage (evaluated in harvested islets 24 h after microporation) was similar to that of islets simply incubated in the presence of the contrast agent (endocytosis). A higher voltage (900–1500 V) in the case of the standard protocol significantly decreased viability of labelled and harvested islets compared to islets labelled by endocytosis (*U* test, *P* < 0.05, marked by “*” in Fig. 4a). Similarly, viability decreased gradually with increasing voltage in the case of the modified protocol; at 800 V and higher the decrease was significant compared to endocytosis (*P* < 0.5, marked by “*” in Fig. 4b).

Percentages of harvested islets after microporation (gain, 100% = number of islets before labelling) were significantly lower in the case of standard procedure compared to endocytosis even at low voltage applied during microporation (*U* test, *P* < 0.05, marked by “×” in Fig. 4a). Contrary to this, gain in the case pre-microporation was comparable to endocytosis at lower voltages. Significant decrease of the gain was observed at pre-microporation using 1000 V only (*U* test, *P* < 0.5, marked by “×” in Fig. 4b).

Both MRI and optical fluorescence imaging proved that collected PIs were efficiently labelled by both microporation procedures (Fig. 4c-f).

Based on ^19^F MR data analysis, the labelling efficiency of microporation using the modified protocol was substantially lower than endocytosis (Fig. 4d). However, a 1-h MR scan still provided a detectable signal from 250 microporated islets.

In contrast, for optical fluorescence imaging (Fig. 4f) the endocytosis provided the lowest signal, albeit not significantly different from the signal of pre-microporated islets due to high data dispersion. High probe content (proved by ^19^F MRI, Fig. 4d) in the case of islets after endocytosis did not lead to correspondingly high optical signal. This discrepancy can be explained by quenching of the optical signal caused by locally high concentration of the probe in the islets. The phenomenon of a decrease of a normalized fluorescent signal with increasing ICG concentration was described by Yuan et al. [41]. Two effects may be responsible for this decrease. Propagation depth of the excitation light in the solution decreases with the increasing ICG concentration. It consequently causes decreasing illumination by the excitation light, and therefore the decay of the emission strength. With increasing concentration increases also re-absorption of emission photons, which contributes to the decay of the emission strength too.

**Fig. 5:**
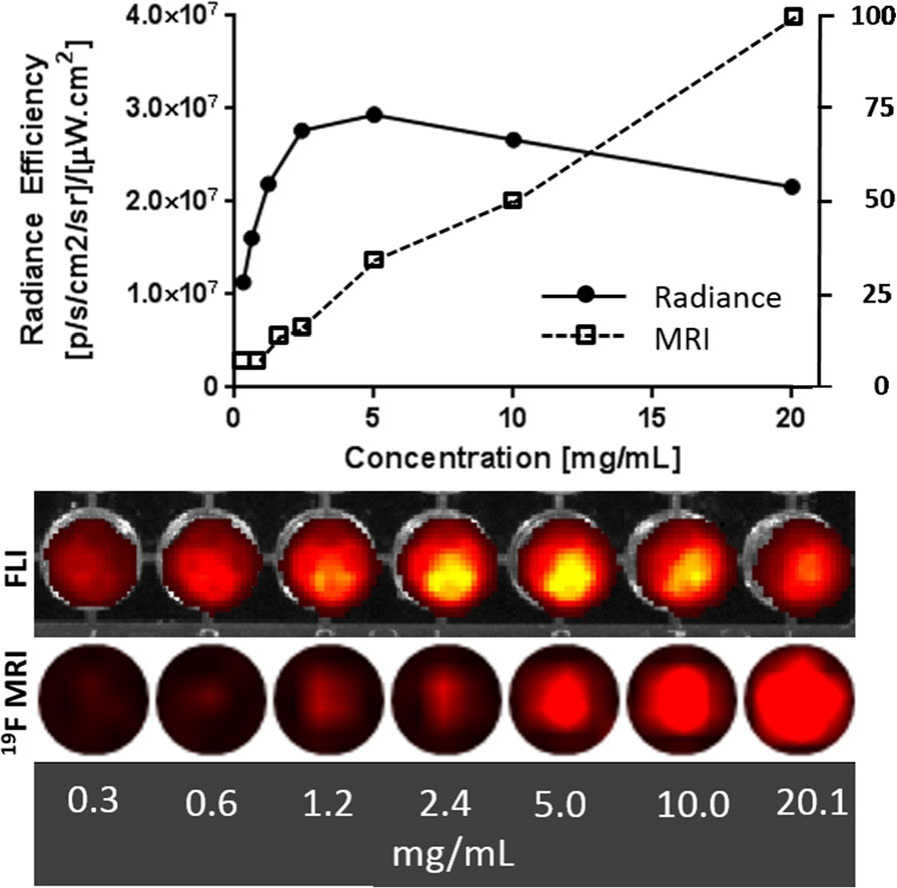
Dependence of the fluorescent signal (*full circles*, *solid line*) and of the ^19^F MRI signal (*empty squares*, *dotted line*) from the nanoparticle suspension on concentration. The fluorescent signal decreased at high probe concentrations due to signal quenching, while the MRI signal linearly increased. Actual fluorescent and MRI images of the test tubes with nanoparticles at different concentrations are presented below the graph

To confirm this, we tested the fluorescence of the probe itself in vitro*.* Figure 5 shows a decrease in fluorescent signal intensity at higher concentrations caused by signal quenching, which corresponds to the curve shapes in [41]. MRI signal increased linearly with increasing concentrations. Both optical and MRI images of the test tubes with different nanoparticle concentrations are shown under the graph.

**Fig. 6:**
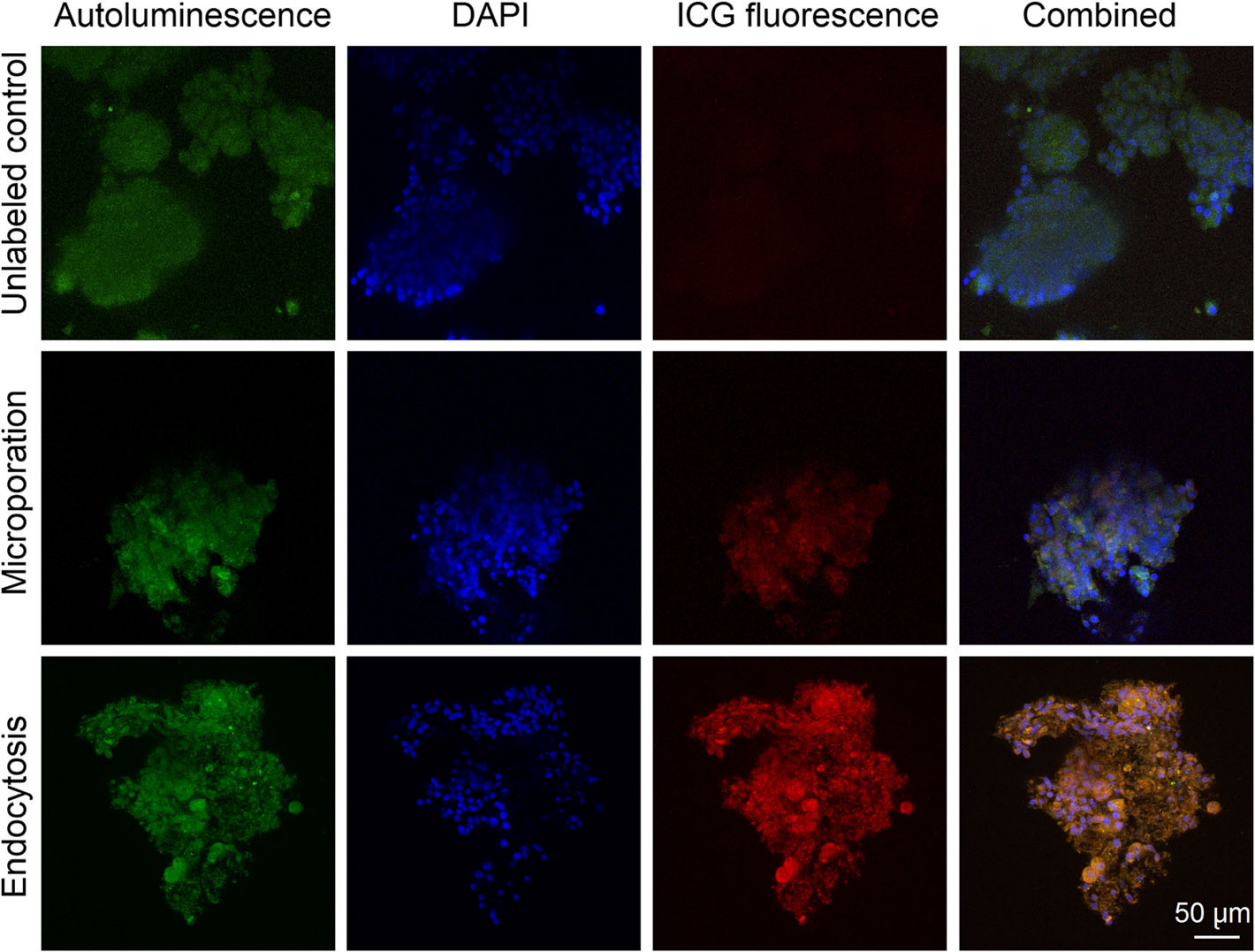
Confocal microscopy of the fixed sliced pancreatic islet samples. *Upper row* – a control sample of unlabelled pancreatic islets; *middle row* – islets labelled by microporation according to the modified protocol at 600 V; *lower row* – islets labelled by endocytosis. Artificial colours represent autoluminescence (*green*), DAPI (*blue*) and the fluorescent signal of ICG bound to nanoparticles (*red*). The far right column shows an overlap of *green*, *blue* and *red* signals. The scale bar represents 50 μm

Confocal microscopy of the fixed pancreatic islets confirmed the presence of fluorescent nanoparticles in islets labelled using microporation or endocytosis (see Fig. 6). Higher fluorescent signals (red) in the case of endocytosis showed higher efficiency of labelling by endocytosis compared to microporation, a finding which is in agreement with MR results.

Our results indicated that standard microporation protocol led to substantial loss of islets during the procedure. Also, higher voltage pulses (>800 V) as well as usage of more than two pulses led to radical impairment of islet viability (Figs. 2a and 3a, b).

Therefore we fixed the optimal settings for pancreatic islet labelling using microporation as follows:

- “pre-microporation”: microporation without nanoparticles with two 20 ms pulses 600 V (field 20 kV/m),
- placing of microporated islets in the medium with suspended nanoparticles (final concentration 23 mg/mL value in the solution) on ice for 10 min,
- transferring islets to a Petri dish containing the medium and 24-h incubation at 37 °C for recovery (incubation may be shorten, if necessary).

This protocol ensures the high gain and viability of the islets as well as reasonably high labelling efficiency.

It should be noted that the protocol was optimized for the nanoparticles used in this study, which have an average diameter of 200 nm and a slight positive charge. Microporation efficiency may substantially differ with different contrast agents.

## Discussion

Pancreatic islet transplantation represents an alternative treatment for Type 1 diabetes, however, in vivo monitoring of the transplant is still a challenge. It requires labelling of islets corresponding to the used imaging method. ^19^F MRI is a highly specific method, albeit with low sensitivity, which is therefore scarcely used [42]. Combination with a more sensitive fluorescent probe seems to be a suitable solution. To ensure sufficient probe content and viability of the islets, labelling strategies should be carefully optimised with respect to the selected probe and complex islet structure.

Although electroporation is widely used for cell labelling, there are only a few reports on pancreatic islet labelling. Tai et al. [32] successfully used electroporation at lower voltages (up to 100 V) for SPIO-based labels. However, in our experience, microporation (which requires a higher voltage due to electrode geometry) is an invasive procedure and may cause cell death using any combination of parameters. In our study, the number of islets decreased substantially after microporation, especially at higher voltages. Moreover, viability was tested on unbroken islets and does not reflect the fact that some islets were completely destroyed or lost during labelling. Also, it should be noted that the procedure itself demands skilful work, since pancreatic islets require 100 μL pipette tips (which are broader) and the islets tend to fall out of the tips quite quickly due to gravity when the pipette is positioned vertically during microporation.

Microporation according to the standard procedure (i.e. in the presence of nanoparticles) was very difficult. The coloured suspension made visual control of the islets impossible during microporation and parts of the pancreatic islets may have been lost in the electrolytic buffer during the procedure. In addition, the nanoparticle suspension was somewhat foamy and air bubbles caused occasional arcing (sparks) in the sample, which harmed the islets. The bubbles are in fact caused by the nanoparticles used. At a certain concentration the suspension becomes foamy due to the hydrophilic surface of the polymer [35]. The nanoparticles probably decrease surface tension and behave like a surfactant. Although visible bubbles were removed before microporation, the removal of microbubbles responsible for sparks during poration is virtually impossible.

The original microporation procedure (voltage application in the presence of nanoparticles) often led to sparks in the solution with islets, resulting in their destruction. The modified protocol (microporation without the nanoparticles and placing the porated islets in the medium with nanoparticles immediately after voltage application) avoided these problems. Incubation of the islets on ice after microporation slowed down the sealing of the membranes and enabled nanoparticle uptake. The outcomes from the labelling according to the modified protocol were more than 2-fold higher.

### Comparing Microporation and Endocytosis

In contrast to microporation, the uptake of the agent by endocytosis was simple, efficient, and did not substantially affect cell viability. Microporation according to the standard protocol may label islets at comparable efficiency, but islet viability is compromised and many islets may be lost during the procedure. Microporation according to the modified protocol ensures higher viability and lower loss of islets. The only comparable setting for microporation in terms of viability was the usage of two low voltage pulses 600 V/20 ms (without nanoparticles – i.e., “pre-microporation”) and incubation in the presence of nanoparticles on ice for 10 min. Brief exposure to nanoparticles might be beneficial in specific applications and the method may be suitable when fast labelling is required. Although labelling under these conditions was not as efficient as 24-h labelling using simple endocytosis being up to 3-fold lower, it is necessary to keep in mind that faster labelling procedures may fit better in clinical or preclinical schedules. 10 min incubation on ice adjusted according to [35] represents a reasonable time interval for sufficient islet labelling and still with no adverse effects on islet viability.

The discrepancy between semi-quantitative evaluation of MR images and optical signals (Fig. 4d and f) also indicates that high labelling efficiency is not necessarily better for optical fluorescence imaging. High label concentrations lead to signal losses due to quenching. High uptake of the probe by endocytosis (confirmed in our experiment by MRI, Fig. 4d, and confocal microscopy, Fig. 6) thus resulted in a similar optical signal (Fig. 4f). We observed signal quenching at higher concentrations in the pure nanoparticle suspension (Fig. 5) manifested by fluorescent signal decrease, while MRI signal increased with increasing concentration. We presume that the average concentration in the medium with labelled PIs was lower. However, the nanoparticles may not have been evenly distributed within the PI pellets, which may have led to signal loss.

Although optical imaging is often presented as a quantitative method, signal quenching makes exact quantitation of the source particles impossible. Nevertheless, it should be noted that under in vivo conditions, islets may be more dispersed than under in vitro conditions, and the effect of quenching may be negligible. Also, quantitation of MR signals from images may be subject to errors and artefacts when taking into account the following hardware and software limitations: use of a surface coil with substantial B_1_ inhomogeneity, low measurement matrix (32 × 32), filtering during post-processing, and Fourier transform, leading to partial signal (and noise) dispersion across the whole image matrix.

## Conclusions

The experiment confirms that microporation – although a fast and effective labelling method and one that is documented in the literature as a treatment for use with PIs – is invasive and may cause substantial harm to islets.

We modified the protocol and optimised microporation parameters to achieve sufficient labelling and minimise viability loss during the procedure. Poration in the presence of nanoparticles was found to be unsuitable for the PLGA nanoparticles used and therefore was replaced by pre-microporation of islets in the buffer without nanoparticles. Moving the islets into the nanoparticle suspension after poration and keeping them on ice for 10 min slowed down the sealing of pores and facilitated the migration of nanoparticles to cells. Subsequent incubation in the medium at 37 °C enabled the pores to be sealed and the recovery of islets.

Compared to endocytosis, the labelling efficiency of microporation was lower despite the modified protocol substantially improving the viability and gain of the method. However, the labelling with pre-poration was very fast.

Microporation may thus be considered a suitable labelling procedure when fast cell labelling is desired.

## Abbreviations

DAPI: Diamino-phenylindole
FBS: Foetal bovine serum
HEPES: Buffer based on (4-(2-hydroxyethyl)-1-piperazineethanesulfonic acid
ICG: Indocyanine green
MR: Magnetic resonance
MRI: Magnetic resonance imaging
PBS: Phosphate buffer saline
PET: Positron emission tomography
PFCE: Perfluoro crown ether
PI: Pancreatic islet
PLGA: Poly(lactic-co-glycolic acid)
SPECT: Single-photon emission computed tomography
